# Identification and Characterization of *Colletotrichum fructicola* and *Colletotrichum siamense* Causing Anthracnose on Luffa Sponge Gourd in China

**DOI:** 10.3390/plants11121537

**Published:** 2022-06-08

**Authors:** Ping Li, Jun-Zi Zhu, Xiao-Gang Li, Jie Zhong

**Affiliations:** 1 Hunan Provincial Key Laboratory for Biology and Control of Plant Diseases and Insect Pests, Hunan Agricultural University, Nongda Road 1, Furong District, Changsha 410128, China; lipinghnau@163.com (P.L.); zjz1608@sina.com (J.-Z.Z.); 2 Hunan Engineering Research Center of Agricultural Pest Early Warning and Control, Hunan Agricultural University, Nongda Road 1, Furong District, Changsha 410128, China

**Keywords:** morphological characterization, multi-locus phylogenetic analysis, *Luffa cylindrica*, *Colletotrichum fructicola*, *Colletotrichum siamense*

## Abstract

Luffa sponge gourd (*Luffa cylindrica*) is an important cucurbitaceous vegetable and is known as the source of loofah. From 2020 to 2021, a leaf disease occurred on luffa leaves in the Hunan Province of China. Symptoms were displayed as oval to irregular chlorotic lesions surrounded by yellow halos. The pathogens were isolated from the affected leaves. According to morphological characterization and molecular identification using multi-locus phylogenetic analysis of the internal transcribed spacer (ITS), actin (*ACT*), chitin synthase (*CHS-1*), glyceraldehyde-3-phosphate dehydrogenase (*GAPDH*), β-tubulin (*TUB2*), and partial mating type (Mat1-2) gene (*ApMAT*) regions, the pathogens were identified as two *Colletotrichum* species: *Colletotrichum fructicola* and *C. siamense*. Koch’s postulates were identified by a pathogenicity test and re-confirmation. To the best of our knowledge, *C. fructicola* and *C. siamense* are two new species associated with luffa sponge gourd anthracnose.

## 1. Introduction

*Luffa cylindrica* (L.) Roem (Luffa sponge gourd), belonging to the Cucurbitaceae family, is an important horticultural crop. This plant is widely cultivated in tropical and subtropical areas globally, including countries in Asia, America, and Africa [1]. The sponge gourd is a commercial crop since it has important vegetable and medicinal values [2,3]. In recent years, luffa sponge gourd planting has had good economic benefits, and the cultivation area is expanding. However, many diseases have been recorded in the luffa sponge gourd. For example, cucurbit downy mildew caused by *Pseudoperonospora cubensis* [4,5], gray mold caused by *Botrytis cinerea* [6,7], Fusarium wilt caused by *Fusarium oxysporum*. sp. luffa [8], stem rot caused by *Sclerotium rolfsii* [9], phytophthora fruit rot caused by *Phytophthora capsica* [10], and fruit rot caused by *Stagonosporopsis cucurbitacearum* [11]. In addition, anthracnose is a common disease in *Cucurbitaceae* crops [12]. *Colletotrichum orbiculare* has been recorded as the pathogen of *L. cylindrica* [13]. However, until now, there has been no reports of other *Colletotrichum* species causing luffa anthracnose.

**Figure 1:**
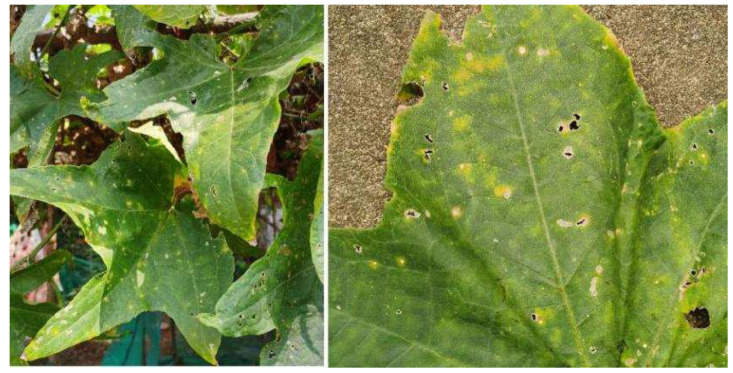
Symptoms of the diseased luffa sponge gourd leaves in field.

In August of 2020 and 2021, we observed the occurrence of a leaf spot disease on sponge gourd in some private cultivation farms in Hunan Province of China. Almost 90% of the surveyed leaves were infected. Disease symptoms mainly occurred in the leaf of sponge gourd, including water soaking, round, light yellow, chlorosis spots, and extended or coalesced to oval or irregular lesions. The spots were brown at the margin and grayish white in the center, with clear yellow halos surrounding the outside. When it was dry, the center of the spots were easily perforated and ruptured (Figure 1). At a later stage, lesions expanded further and caused leaf blight and wither. In this study, the pathogen responsible for the disease was determined by examination of its morphological and molecular characteristics, and through pathogenicity testing to fulfill Koch’s postulates.

## 2. Results

### 2.1. Fungal Isolation

A total of 10 fungal isolates were obtained from 10 affected sponge gourd leaves that were collected from 10 cultivation farms in Changsha of Hunan Province. The information of the isolates are shown in Table 1. Based on the colony morphology, the fungal strains were primarily identified as *Colletotrichum* species. Four isolates, which could be putatively divided into two types, were selected for further morphological and molecular identification.

### 2.2. Morphological Characterization

**Figure 2:**
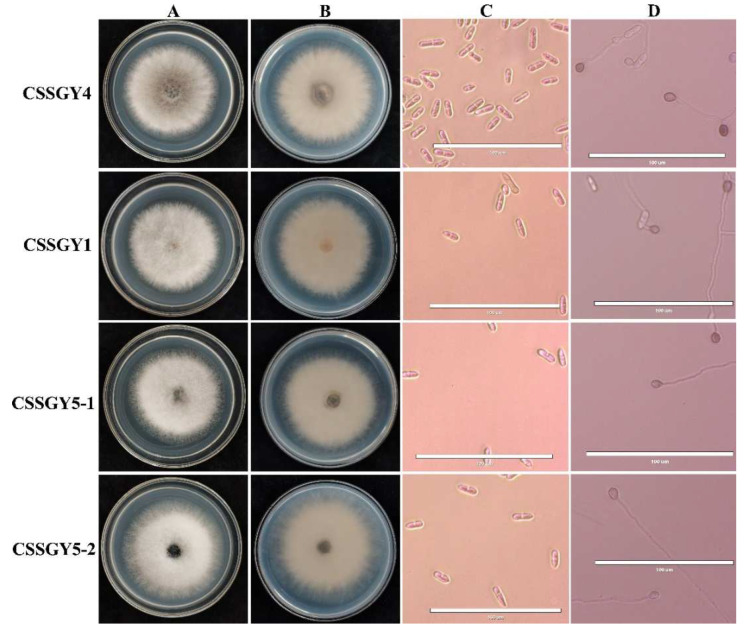
Morphological characteristics of colonies, conidia, and appressoria of the *Colletotrichum* isolates isolated from luffa sponge gourd. (**A**) Colony morphology of the positive sides of the fungal strains CSSGY4, CSSGY1, CSSGY5-1, and CSSGY5-2 cultured on potato dextrose agar (PDA). (**B**) Reverse sides on PDA. (**C**) Microscopic examination of the conidia. (**D**) Microscopic examination of the appressoria. Scale bar = 100 μm.

When cultured on potato dextrose agar (PDA) medium, CSSGY1, CSSGY5-1, and CSSGY5-2 were white and gradually turned grey white, containing dense aerial mycelium (Figure 2A,B). For strains CSSGY1, CSSGY5-1, and CSSGY5-2, conidia were hyaline, unicellular, aseptate, long elliptic to cylindrical, and measured 12.24–28.57 × 4.08–8.29 μm. Appressoria were brown, nearly elliptical or irregular, and measured 6.12–12.24 × 4.08–8.16 μm. For strain CSSGY4, the colony was greyish green to brown. Conidia were hyaline, unicellular, and long cylindrical, with an average size of 14.29–24.49 μm × 4.08–8.16 μm. Appressoria were brown to dark black, ovoid to slightly irregular, and 6.12–10.20 × 4.08–8.16 μm (Figure 2C,D). The features of conidia and appressoria of the four strains are described in Table 2. A combination of the morphological characteristics and phylogenetic analysis confirmed that CSSGY1, CSSGY5-1, and CSSGY5-2 strains belonged to *C. siamense* while CSSGY4 belonged to *C. fructicola.*

### 2.3. Molecular Identification

For molecular verification, the internal transcribed spacer (ITS), actin (*ACT*), chitin synthase (*CHS-1*), glyceraldehyde-3-phosphate dehydrogenase (*GAPDH*), β-Tubulin 2 (*TUB2*), and partial mating type (Mat1-2) gene (*ApMAT*) sequences of four representative strains, CSSGY1, CSSGY4, CSSGY5-1, and CSSGY5-2, were amplified and sequenced. All obtained sequences were deposited in GenBank, with the accession numbers listed in Table 3. BLASTn searches showed that the ITS of CSSGY1, CSSGY4, CSSGY5-1, and CSSGY5-2 was 99.46% to 100% identical with the *C. gloeosporioides* isolates while the *GAPDH*, *ACT*, *CHS-1*, *TUB2*, and *ApMAT* sequences of the CSSGY1, CSSGY5-1, and CSSGY5-2 were most similar to the *C. siamense* strains, with identities ranging from 97.94% to 100%. For CSSGY4, the *GAPDH*, *ACT*, *CHS-1*, *TUB2*, and *ApMAT* sequences had a 99.59% to 100% identity with the corresponding sequences of *C. fructicola* strains.

**Figure 3:**
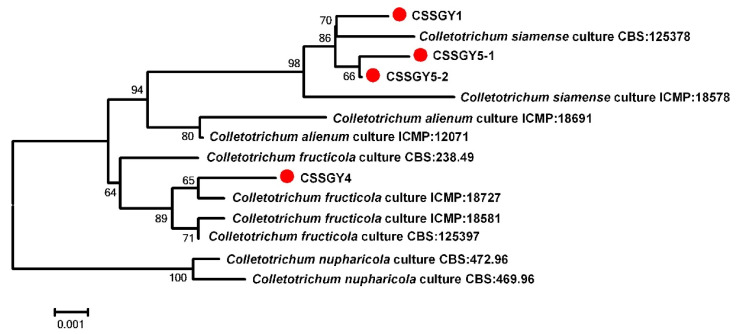
Construction of a phylogenetic tree based on concatenated sequences of the internal transcribed spacer (ITS), actin (*ACT*), chitin synthase (*CHS-1*), glyceraldehyde-3-phosphate dehydrogenase (*GAPDH*), β-tubulin (*TUB2*), and partial mating type (Mat1-2) gene (*ApMAT*) regions, using the neighbor-joining method. Notes in the branches indicate the bootstrap values supporting the branches that were calculated from the bootstrap test of 1000 replicates. The isolates CSSGY1, CSSGY4, CSSGY5-1, and CSSGY5-2 were marked with red dots. The scale bar indicates the number of substitutions at each position. The sequence information of the *Colletotrichum* species used for phylogenetic analysis is shown in Table 3.

For further phylogenetic analysis, a neighbor-joining phylogenetic tree based on concatenated datasets of ITS, *ACT*, *CHS-1*, *GAPDH*, *TUB2*, and *ApMAT* was constructed, which included the 4 strains and 10 referenced *Colletotrichum* strains. Phylogenetic analysis revealed that the strains CSSGY1, CSSGY5-1, and CSSGY5-2 were clustered with the *C. siamense* clade, including the type strain CBS: 125378, while the strain CSSGY4 was grouped with the *C. fructicola* clade, which was consistent with the homology search results that were conducted using BLASTn (Figure 3).

### 2.4. Pathogenicity Test

**Figure 4:**
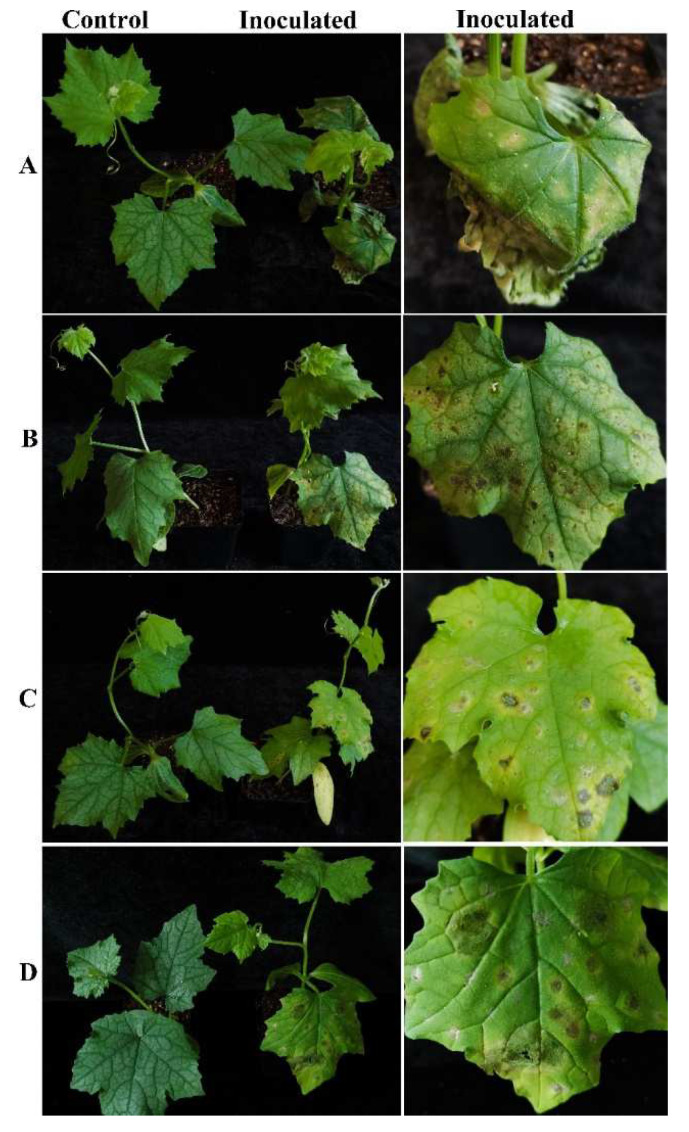
Pathogenicity test of strains CSSGY1, CSSGY4, CSSGY5-1, and CSSGY5-2 on luffa sponge gourd plants. (**A**–**D**) Symptoms of potted sponge gourd plants that were artificially inoculated with *Colletotrichum* isolates of (**A**) CSSGY1, (**B**) CSSGY4, (**C**) CSSGY5-1, and (**D**) CSSGY5-2. The left and right plants indicate the control and inoculated plants, respectively. After 5 days of inoculation, all the inoculated sponge gourd leaves developed symptoms. No obvious symptoms developed on the control plants.

The four representative isolates were selected for a pathogenicity test. Luffa potted sponge gourd plants were in vivo inoculated with conidial suspensions. Five days after inoculation, all the inoculated sponge gourd leaves developed symptoms, showing yellow halos in the inoculated sites. Later, leaf spots occurred, which were enlarged, and in some case were perforated, which was similar to those observed in the field. No symptoms were observed on the control plants (Figure 4). All four strains were pathogenic to the luffa sponge gourd plants and caused similar symptoms. The pathogenic fungi were recovered from the inoculated symptomatic leaves, and identified by morphological observation and molecular sequencing using the methods described above, thus fulfilling Koch’s postulates.

## 3. Discussion and Conclusions

In this study, the fungal pathogens of the leaf-spot-infected luffa sponge gourd were isolated and identified by cultural and microscopic examination; phylogenetic analysis was carried out using the concatenated datasets of ITS, *ACT*, *CHS-1*, *GAPDH*, *TUB2*, and *ApMAT*; and pathogenicity tests were carried out on potted luffa sponge gourd plants. The pathogens were confirmed to be two *Colletotrichum* species: *C. siamense* and *C. fructicola*. Therefore, this disease in luffa sponge gourd was identified as anthracnose. To our knowledge, this is the first report of *C. siamense* and *C. fructicola* causing anthracnose in luffa sponge gourd. The identification of this new disease and its causal agent provides an important basis for the diagnosis and control of diseases in luffa sponge gourd.

*Colletotrichum* species is a kind of fungal group that cause diseases in a wide range of plant hosts. Some species in the *Colletotrichum* genera are difficult to distinguish using sole morphological observation and single gene locus analysis; thus, multi-gene phylogenetic analysis is considered necessary for the identification of individual *Colletotrichum* isolates [14]. In our study, based on the concatenated ITS, *ACT*, *CHS-1*, *GAPDH*, *TUB2*, and *ApMAT* six-gene phylogenetic analysis, the four representative isolates that were pathogenic to luffa sponge gourd were classified into two *Colletotrichum* species: *C. siamense* and *C. fructicola*. This is the first time these species have been recorded as pathogens that cause luffa anthracnose, which is another aspect that broadens our understanding of various plant diseases caused by the two *Colletotrichum* species.

*C. fructicola* is a species established from the *C. gloeosporioides* species complex. It has a worldwide distribution with a wide host range, including some important fruit crops, such as apple, pear, chili and strawberry, and other cash crops and vegetables, such as oil tea [15], tea [16], and chili [17]. The disease of particular concern caused by *C. fructicola* is the *Glomerella* leaf spot of the apple, which could lead to severe defoliation of the infected plants [18,19]. *C. siamense* is also in the *C. gloeosporioides* species complex, which was first recorded on coffee berries [20]. *C. siamense* has been reported as a worldwide pathogen of anthracnose on a variety of economically important plants, including fruits, ornamental plants, and vegetables, such as the mango, custard apple, papaya, tea plants, chili pepper, persimmon, orange, avocado, guava, rubber plant, and jasmine [21,22,23,24,25,26]. Until now, reports of disease caused by *C. fructicola* and *C. siamense* in cucurbitaceous vegetables have rarely been reported.

The anthracnose disease can damage leaves, often leading to wilting and defoliation. Therefore, it might seriously affect the photosynthesis of the plants, which in turn influences the yield of luffa sponge gourd fruit. Since *C. siamense* and *C. fructicola* also have a relatively wide host range, it might be possible that the pathogen of luffa sponge gourd can also infect other vegetables grown nearby. In this study, anthracnose of luffa sponge gourd occurred in almost 90% of the plants. Therefore, to mitigate further possible damage caused by this disease, studies in global prevention and management strategies should be conducted. Considering food and environmental safety, resistant breeding might be an ideal method to control this disease. In addition, the development of molecular detection methods is also important for early diagnosis and prevention of the disease.

In conclusion, the causal pathogens of emerging anthracnose on luffa sponge gourd were identified and characterized. Fungal isolates were isolated and identified by morphological characteristics and multi-gene sequencing and phylogenetic analysis. The pathogenicity of the representative isolates was tested. Identification of these pathogens might provide important insights for the diagnosis and management of disease on luffa sponge gourd.

## 4. Materials and Methods

### 4.1. Sample Collection and Fungal Isolation

From August to September 2020 and 2021, luffa sponge gourd leaves that exhibited visible leaf spots were collected from different vegetable gardens in Changsha, the Hunan Province of China. Symptoms of the diseased leaves were observed and recorded. Pieces of small sections in approximately 5 × 5 mm fragments were cut off from the junction of lesions, disinfected with 70% alcohol for 30 s, 0.1% mercuric chloride for 45 s, rinsed with sterile distilled water 3 times, and then placed on potato glucose agar medium (PDA) and cultured at 28 °C in the dark. Hyphal tips developed from the sample pieces were picked and transferred onto fresh PDA plates and incubated at 28 °C. Single-spore isolation was conducted to purify the fungal cultures. All single-spore cultures were stored at 4 °C in a refrigerator. 

### 4.2. Morphological and Cultural Characterization

For morphological examination, mycelial plugs (8 mm diam) of the fungal strains were plated on PDA and cultured at 28 °C. After 7 days, the colony characteristics of the morphology and color were observed. The morphology of the conidia and appressoria was observed at 400× magnification using an optical microscope (life technology, EVOS)™ XL core imaging system). The length and width of the conidia and appressoria were determined by measuring about 50 randomly selected structures each. 

### 4.3. Molecular Identification

Genomic DNA was extracted from mycelium using the CTAB method as described previously [27] and used as templates for PCR amplification. The ITS, *ACT*, *CHS-1*, *GAPDH*, *TUB2*, and *ApMAT* gene regions were amplified using the primers ITS4/ITS5 [27,28], ACT512F/ACT783R [23], CHS-79F/CHS-345R [29], GDF/GDR [30], T1/Bt2b [15], and AMF1/AMR1 [31], respectively. The polymerase chain reaction (PCR) was performed in a 25 μL reaction system, including 1 μL genomic DNA, 12.5 μL PCR Master Mix, 1 μL (10 mM) of each primer, and 9.5 μL of sterile distilled ddH_2_O. The PCR procedure consisted of initial denaturation at 95 °C for 4 min, followed by 34 cycles of 95 °C for 30 s (denaturation), 56 to 60 °C for 30 s (annealing), 72 ℃ for 45 s (extension), and a final extension of 72 °C for 5 min. PCR products were obtained using 1% agarose gel electrophoresis. The PCR products were sequenced by Shanghai Sangon Company (China) using the dideoxy termination method. All the obtained ITS, *ACT*, *CHS-1*, *GAPDH*, *TUB2*, and *ApMAT* sequences were submitted to the GenBank database and compared with the National Biotechnology Information Center (NCBI) database through BLASTn [32]. 

The ITS, *ACT*, *CHS-1*, *GAPDH*, *TUB2*, and *ApMAT* sequences of our isolates and other similar *Colletotrichum* species were selected for phylogenetic analysis. The concatenated sequences of the ITS, *ACT*, *CHS-1*, *GAPDH*, *TUB2*, and *ApMAT* loci were aligned with ClustalX [33] and subjected to phylogenetic tree construction using the NJ method in MEGA 6, with the bootstrap values calculated by 1000 replications [34].

### 4.4. Pathogenicity Assay

To fulfill the Koch’s postulates, the pathogenicity tests of the representative strains were carried out by in vivo inoculation in potted sponge gourd plants with conidial suspensions. The four strains were cultured on PD liquid medium at 28 °C in a shaker incubator (180 rpm/min) for 4 days, filtered with 4 layers of sterile gauze to harvest the conidial suspensions, and then they were adjusted to a final concentration of 1 × 10^5^ spores/mL by a hemocytometer. Leaves of the luffa sponge gourd plants were disinfected with 70% alcohol. Inoculation was carried out by spraying 5 mL of conidial suspension on each plant while sterile water was inoculated on control plants using the same procedure. All the inoculated plants were placed in plastic chamber with 95% relative humidity at 26 °C under a 16/8 h light/dark cycle. All the inoculums were observed daily for symptom progression. The experiments were repeated twice, with three potted plants inoculated for each strain. 

To confirm Koch’s postulates, the pathogen fungal strains were re-isolated from the experimentally symptomatic leaves and identified according to the morphological and molecular characteristics as mentioned above. 

### 4.5. Data Analysis

Statistical analyses were conducted using Statistical Package for Social Sciences (SPSS) (Version 22.0 for Windows, IBM Corp., Armonk, NY, USA). Analysis of variance (ANOVA) on the conidial and appressoria length and width was performed. Means were compared using the least significant difference test at a significance level of *p* = 0.05.

## Figures and Tables

**Table 1:** The information of the isolates from luffa sponge gourd.

Specimen Number	Host	Varieties	Geographic Origin (City, Province)
SGY1	*Luffa cylindrica*	Zhenli	Changsha, Hunan
SGY2	*Luffa cylindrica*	Zhenli	Changsha, Hunan
SGY3	*Luffa cylindrica*	Zhenli	Changsha, Hunan
SGY4	*Luffa cylindrica*	Jingpinbaili	Changsha, Hunan
SGY5-1	*Luffa cylindrica*	Jingpinbaili	Changsha, Hunan
SGY5-2	*Luffa cylindrica*	Jingpinbaili	Changsha, Hunan
SGY6	*Luffa cylindrica*	Jingpinbaili	Changsha, Hunan
SGY7-1	*Luffa cylindrica*	Changsharousigua	Changsha, Hunan
SGY7-2	*Luffa cylindrica*	Changsharousigua	Changsha, Hunan
SGY8	*Luffa cylindrica*	Changsharousigua	Changsha, Hunan

**Table 2:** Morphological characteristics of *Colletotrichum* strains isolated from luffa sponge gourd.

Strain Name	Conidia Size (μm)		Appressoria Size (μm)	
	Length Average ± SD ^a^	Width Average ± SD ^a^	Length Average ± SD ^a^	Width Average ± SD ^a^
CSSGY1	23.57 ± 4.84 ^a^	6.22 ± 1.23 ^a^	8.47 ± 1.78 ^a^	6.12 ± 1.41 ^a^
CSSGY4	16.33 ± 2.63 ^a^	6.12 ± 1.17 ^b^	8.16 ± 1.31 ^b^	6.12 ± 0.80 ^a^
CSSGY5-1	15.31 ± 2.61 ^a^	5.71 ± 1.42 ^c^	8.88 ± 1.20 ^c^	6.12 ± 0.91 ^a^
CSSGY5-2	15.31 ± 2.43 ^a^	5.71 ± 2.25 ^d^	9.11 ± 1.06 ^d^	7.06 ± 1.06 ^b^

^a^ SD means standard deviation. Values within the same column followed by different letters mean that they are significantly different based on variance with the least significant difference test at *p* = 0.05.

**Table 3:** Information on the GenBank accession numbers of the *Colletotrichum* species used for phylogenetic analysis.

Species	Isolate/Strain	GenBank Acession Number					
		ITS	*ACT*	*CHS*	*GAPDH*	*TUB2*	*ApMAT*
*Colletotrichum siamense*	CBS 125378	MH863512	JX009441	JX009875	JX010019	JX010410	KP703513
*Colletotrichum siamense*	ICMP 18578	JX010171	FJ907423	JX009865	JX009924	JX010404	JQ899289
*Colletotrichum siamense*	CSSGY1	ON428107	ON454464	ON454476	ON454480	ON454468	ON454472
*Colletotrichum siamense*	CSSGY5-1	ON428108	ON454466	ON454478	ON454482	ON454470	ON454474
*Colletotrichum siamense*	CSSGY5-2	ON428109	ON454467	ON454479	ON454483	ON454471	ON454475
*Colletotrichum alienum*	ICMP 18691	JX010217	JX009580	JX009754	JX010018	JX010385	
*Colletotrichum alienum*	ICMP 12071	JX010251	JX009572	JX009882	GU174553	JX010411	KC888927
*Colletotrichum fructicola*	ICMP 18727	JX010179	JX009565	JX009812	JX010035	JX010394	
*Colletotrichum fructicola*	ICMP 18581	JX010165	FJ907426	JX009866	JX010033	JX010405	JQ807838
*Colletotrichum fructicola*	CBS 125397	MH863502	JX009581	JX009874	JX010032	JX010409	
*Colletotrichum fructicola*	CSSGY4	ON428106	ON454465	ON454477	ON454481	ON454469	ON454473
*Colletotrichum nupharicola*	CBS:472.96	JX010188	JX009582	JX009836	JX010031	JX145226	JX145320
*Colletotrichum nupharicola*	CBS 469.96	JX010189	JX009486	JX009834	JX009836	JX010399	

## Data Availability

All sequence data are available in NCBI GenBank following the accession numbers in the manuscript.
